# THE LIFE COURSE PERSPECTIVES OF WOMEN AGING WITH SCHIZOPHRENIA: CONCERNS WITH HEALTHY AGING

**DOI:** 10.1093/geroni/igad104.0683

**Published:** 2023-12-21

**Authors:** Veronica Garcia Walker

**Affiliations:** UT at Austin, Austin, Texas, United States

## Abstract

Multiple semi-structured interviews were conducted between August 2021- June 2022 to obtain life course insights of women aging with schizophrenia (N=14). 2 (14%) identified as Hispanic; 5 (36%) as African American, 7 (50%) as non-Hispanic White. Findings: 1) Getting Old Has Made it Worse, indicating decrease in functioning related to aging 2); I Need Meds to Live a Healthier Life, indicating a need for access to health care to address age related health conditions; 3) I’m Afraid to Die, indicating worry expressed as death becomes more imminent; 4) Who’s Going to Help an Old Lady Like Me?, indicating a need for assistance to manage health conditions associated with aging. These findings support the need for experts that are considerate, have good communication, are genuine, and trustworthy in gerontology to provide important care for persons to ensure long, high quality lives for people with severe mental illness.

